# Identification of functional DNA variants in the constitutive promoter region of *MDM2*

**DOI:** 10.1186/1479-7364-6-15

**Published:** 2012-09-01

**Authors:** Marie-Eve Lalonde, Manon Ouimet, Mathieu Larivière, Ekaterini A Kritikou, Daniel Sinnett

**Affiliations:** 1 Division of Hematology-Oncology, Research Center, Sainte-Justine Hospital, 3175 Chemin de la Cote-Sainte-Catherine, Montreal, H3T 1C5, Canada; 2 Department of Pediatrics, Faculty of Medicine, University of Montreal, Montreal, QC H3T 1C5, Canada

**Keywords:** MDM2, SNP, Promoter analysis, Functional validation, Site-directed mutagenesis

## Abstract

Although mutations in the oncoprotein murine double minute 2 (MDM2) are rare, *MDM2* gene overexpression has been observed in several human tumors. Given that even modest changes in MDM2 levels might influence the p53 tumor suppressor signaling pathway, we postulated that sequence variation in the promoter region of *MDM2* could lead to disregulated expression and variation in gene dosage. Two promoters have been reported for *MDM2*; an internal promoter (P2), which is located near the end of intron 1 and is p53-responsive, and an upstream constitutive promoter (P1), which is p53-independent. Both promoter regions contain DNA variants that could influence the expression levels of *MDM2*, including the well-studied single nucleotide polymorphism (SNP) SNP309, which is located in the promoter P2; i.e., upstream of exon 2. In this report, we screened the promoter P1 for DNA variants and assessed the functional impact of the corresponding SNPs. Using the dbSNP database and genotyping validation in individuals of European descent, we identified three common SNPs (−1494 G > A; indel 40 bp; and −182 C > G). Three major promoter haplotypes were inferred by using these three promoter SNPs together with rs2279744 (SNP309). Following subcloning into a gene reporter system, we found that two of the haplotypes significantly influenced *MDM2* promoter activity in a haplotype-specific manner. Site-directed mutagenesis experiments indicated that the 40 bp insertion/deletion variation is causing the observed allelic promoter activity. This study suggests that part of the variability in the *MDM2* expression levels could be explained by allelic p53-independent P1 promoter activity.

## Introduction

The p53 tumor suppressor has a key role in orchestrating cellular responses to various types of stresses, including DNA damage and oncogene activation with apoptosis, cell-cycle arrest, senescence, DNA repair, cell metabolism, or autophagy [1,2]. Malfunction and mutations of *p53* have been found in most human cancers, leading to a deregulated p53 activity that allows cells to proliferate and survive [3]. The activity of p53 is regulated by many proteins, and one of the most extensively studied regulators of p53 is the murine double minute 2 (MDM2) oncoprotein. MDM2 can regulate p53 activity in different ways and even modest modifications of *MDM2* levels can affect the p53 pathway [4]. Firstly, MDM2 directly binds to the p53 transactivation domain, thus inhibiting its transcriptional activity. Secondly, MDM2 promotes ubiquitin-dependent proteasomal degradation of p53 by functioning as an E3 ubiquitin ligase [5,6]. Finally, MDM2 shuttles p53 out of the nucleus to the cytoplasm of the cell, promoting the degradation of p53. Importantly, MDM2 forms a negative-feedback loop in regulating p53 activity, in which p53 induces transcription of *MDM2*, and, in turn, the MDM2 protein inhibits p53 activity (reviewed by Momand et al. [7]).

Although mutations in *MDM2* are rare, *MDM2* overexpression is observed in a number of human tumors due to various mechanisms including gene amplification [8-10] and increased transcription [11,12]. *MDM2* overexpression predisposed transgenic mice to spontaneous tumor formation [13] and therefore, overexpression of *MDM2* may substitute for inactivating mutations in p53 [9]. Because MDM2 is an important negative regulator of p53 activity, overexpression of *MDM2* can result in the inhibition of p53-mediated-transcriptional activation, thereby promoting human carcinogenesis.

Functional sequence variants in promoter regions can lead to variable gene expression levels [14,15]; single nucleotide polymorphisms (SNPs) in promoters of genes implicated in DNA-damage responses and apoptosis could have an impact in an individual's susceptibility to develop cancer [16-21]. Because MDM2 is a key component of the p53-mediated DNA-damage response, promoter SNPs in this gene might influence this highly regulated pathway by modifying cellular MDM2 protein levels [22]. The *MDM2* gene has a basal promoter (P1) and an alternative promoter (P2) starting in the intron 1 [23]. The promoter P2 contains a p53-responsive element and has been shown to regulate *MDM2* levels in stressed cells, whereas the promoter P1 functions mainly in a non-stressed environment [23,24]. The rs2279744 (SNP309) in the intronic *p53*-responsive promoter of the *MDM2* gene has been shown to increase the affinity of the transcriptional activator Sp1, resulting in higher levels of *MDM2* mRNA and protein. This SNP has been shown to attenuate apoptotic activity and accelerate tumor formation [22,25-27]. Several studies have reported associations between rs2279744 and the risk of different types of cancer [28-30]; however, this association has not always been confirmed [31-33]. In an attempt to obtain a more complete view of the *MDM2* promoters, we determined the SNP content and the haplotype structure of the constitutive P1 promoter. Here, we show that distinct P1 promoter haplotypes can influence the p53-independent promoter activity in an allele-specific manner.

## Methods

### SNP discovery in *MDM2* proximal promoter region

The initial search for promoter SNPs (pSNPs) in *MDM2* proximal promoter defined as 2.0 kb upstream of the transcription start site was done using the dbSNP database (build 128) [34]. Seven SNPs were selected for genotyping in a panel of 91 individuals of Western European descent. The Institutional Review Board approved the research protocol and informed consent was obtained from all participants. The corresponding promoter region was amplified in one polymerase chain reaction (PCR) fragment in a 50μL reaction volume, using the following conditions: 20 pmole of 5^′^AAAGCCCAAATTTCCTTGCT3^′^ (forward) and 5^′^CTCCATCTTTCCGACACACA3^′^ (reverse) primers, 2 mM MgCl_2_, 0.2 mM deoxynucleoside triphosphates (dNTPs), 1× Fast Start Taq DNA polymerase buffer and GC rich buffer, 2U Fast Start Taq DNA polymerase (Roche Diagnostics, Laval, Canada) and 15 ng of genomic DNA. The PCR program was 95°C for 3 min; 10 cycles with a denaturation at 95°C for 15 s; annealing at 55–50°C (each cycle decreases by 0.5°C) for 20 s and elongation at 68°C for 2 min; followed by 25 cycles at 50°C for annealing. The amplicons were dot-blotted in duplicate on a nylon membrane and were hybridized with allele-specific oligonucleotides (ASOs) as previously described [35]. Oligonucleotide probes specific for each promoter SNP were used for ASO analysis and are available upon request. A 40 bp insertion/deletion (indel) polymorphism was genotyped by amplification of a 260 bp fragment containing the indel region followed by electrophoresis of the resulting amplicons on a 3% agarose gel to detect one (homozygous) or two bands (heterozygous). PCR conditions were as follows: 20 pmole of 5^′^TTTCCTTTCTGGTAGGCTGG3^′^ (forward) and 5^′^CACCTACTTTCCCACAGAGA3^′^ (reverse) primers, 1.5 mM MgCl_2_, 0.2 mM dNTPs, 1× Fast Start Taq DNA polymerase buffer and GC rich buffer, 1U Fast Start Taq DNA polymerase (Roche Diagnostics, Laval, Canada), and 15 ng of genomic DNA. The PCR program was 95°C for 3 min; 32 cycles with a denaturation at 95°C for 30 s; annealing at 52°C for 30 s; and elongation at 72°C for 20 s. Hardy-Weinberg equilibrium was tested with a χ^2^ test for goodness of fit. Haplotypes were generated by PHASE software (version 2; University of Washington, Seattle, WA, USA) [36].

### Gene reporter assays and site-directed mutagenesis

#### Constructs

The two major promoter haplotypes (approximately 2.0 kb region) were amplified from genomic DNA of known homozygous individuals and cloned individually in the promoterless pGL3basic Firefly luciferase vector (Promega Corp., Fitchburg, WI, USA) using the Gateway Technology (Invitrogen Corporation, Carlsbad, CA, USA). Specific mutations were introduced by site-directed mutagenesis (Quickchange multi site-directed mutagenesis kit, Stratagene from Agilent Technologies, Santa Clara, CA, USA) according to the manufacturer's instructions. Clones chosen for transfection were sequenced to confirm the presence of the SNPs and then purified using the Qiagen plasmid mini kit (Qiagen Company, Toronto, Canada) prior to transfection.

#### Transfection

The resulting constructs were used to transiently transfect three cell lines (HeLa, HepG2, and JEG3) using lipofectamine reagent according to the manufacturer's protocol (Invitrogen). Constructs (99 ng) and SV40-driven (1 ng) *Renilla* luciferase cytomegalovirus (CMV) immediate early enhancer/promoter region (pRL-CMV) (ratio 100:1) were co-transfected to control transfection efficiency. The pGL3basic promoterless plasmid (Promega) was used as a negative control and the pGL3SV40 plasmid (Promega) was used as a positive control. The transfected cells were plated in 96-well plates with approximately 6 × 10^4^ cells per well. The cells were harvested 24 h following transfection, and luciferase reporter gene activity was measured with dual-luciferase reporter assay system (Promega) in a SpectraMax 190 luminometer according to the manufacturer's protocol (Molecular Devices, LLC, Sunnyvale, CA, USA). Firefly luciferase activities of the allelic constructs were normalized using the *Renilla* luciferase pRL-CMV activity. The results were expressed as the ratio of Firefly luciferase activity divided by the pRL-CMV internal control activity and expressed as relative luciferase (means ± standard deviation) of four replicates. Three independent experiments were carried out for each cell line. Statistical analyses were performed using unpaired Student's *t* test to determine *p* values. Global *p* value is calculated with Fischer's inverse Chi-squared test [37].

### *In silico* predictions of putative TFBS

MatInspector program from Genomatix Software GmbH (Bayerstrasse, Munich, Germany, http://www.genomatix.de) was used to determine the presence of putative binding sites for known transcription factors. The predicted gain or loss of putative transcription factor binding site (TFBS) due to a given SNP was determined by the optimized matrix threshold as defined in the MatInspector program.

## Results

**Figure 1:**
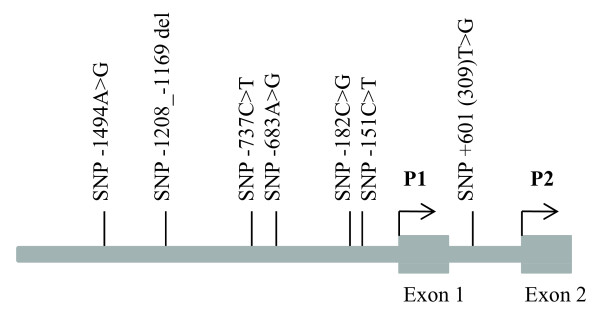
**Schematic illustration of *MDM2* basal (P1) and internal (P2) promoters.** The promoter positions were numbered with respect to the first nucleotide of the first exon as +1, and the nucleotide immediately upstream as −1. The positions of the investigated promoter SNPs are indicated.

**Figure 2:**
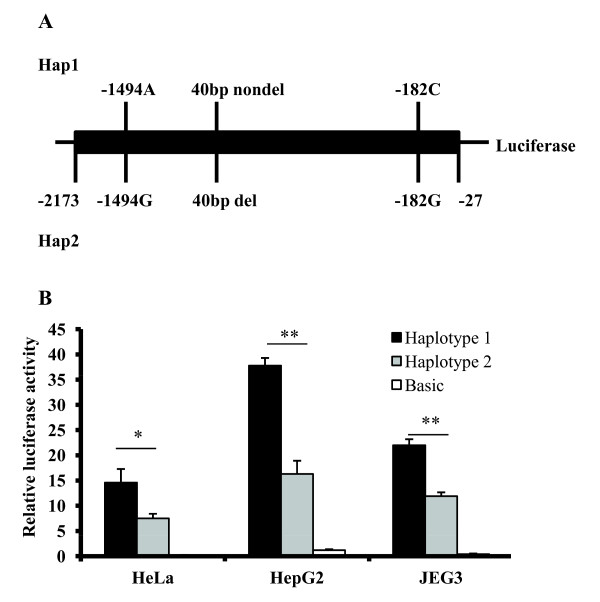
**Gene reporter assays to evaluate the functional impact of the most frequent *MDM2* basal promoter haplotypes.** (**A**) Schematic representation of constructs tested for luciferase gene reporter assays in pGL3 basic vector. Haplotypes H1A and H1B are identical when excluding the position +309 T > G (see Table 2). (**B**) Relative luciferase activity of *MDM2* promoter haplotypes was measured following transient transfection in HeLa, HepG2 and JEG3 cells. The empty promoterless pGL3 basic vector was used as negative control. Results are expressed in a ratio of Firefly/*Renilla* activity multiplied by 100. Promoter haplotype H2 was used as reference against which relative expression was compared. Haplotype H1 showed significantly higher expression levels across all three cell lines. The *p* values are calculated from four replicates with unpaired student's *t* test. Significant differences are marked with an asterisk (*p < 3 × 10^−3^; **p < 8 × 10^−6^).

The search for SNPs in the constitutive P1 promoter of *MDM2* led to the identification of eight pSNPs, including a 40 bp indel (see Table 1). In addition to these pSNPs, we included the well-studied rs2279744 located in the P2 promoter for haplotype analysis (see Figure 1 for a schematic representation of *MDM2* promoters). By genotyping a panel of 91 unrelated Western Europeans, we found four pSNPs (−1494 G > A, rs1144944; indel 40 bp, rs3730485; −182 C > G, rs937282; and SNP309/601 T > G, rs2279744) to be polymorphic. For rs2279744 the observed minor allele frequency of 35% was similar to the one previously reported for Caucasians [38]. Among the five non-polymorphic SNPs, both −1166 T > G (rs2904506) and −1164 C > G (rs3930427) are located in the 40 bp indel sequence thus creating in some individuals a near identical (except for 2 bps) tandem duplication. Therefore, individuals carrying the deletion behave like they have different alleles at these two positions. Because single variants might not be sufficient to capture the genetic variability relative to a given phenotype, we constructed haplotypes using all four polymorphic pSNPs. Based on these data, we estimated haplotype phase and the corresponding frequencies (Table 2). The three most common promoter haplotypes (1A, 1B, and 2) represented 92.3% of the observed haplotypes in Europeans. Haplotypes 1A and 1B differ at rs2279744, whereas haplotype 2 differs at all four positions (Figure 2a). To evaluate the extent of linkage disequilibrium between the SNPs studied, we measured *D*^′^ and *R*^2^; these values between rs1144944 and rs937282 are 0.977 and 0.934, respectively, and 0.968 and 0.471 between these two SNPs and rs2279744. This indicates that rs2279744 (SNP309) is tightly linked with the P1 promoter's variants. 

**Table 1:** **List of the SNPs found in dbSNP database for *MDM2 *promoter **

**Rs number**^**a**^	**Position**^**b**^	**SNPs ID**^**c**^	**MAF**^**d**^
rs1144944	g.67,486,752 G > A	−1494	25%
rs3730485	g.67,487,038_67,487,077del	40 bp indel (−1208 to −1169)	37%
rs2904506	g.67,487,080 T > G	−1166	-
rs3930427	g.67,487,082 C > G	−1164	-
rs3730486	g.67,487,509 C > T	−737	0%
rs3730487	g.67,487,563A > G	−683	0%
rs937282	g.67,488,064 C > G	−182	50%
rs3730491	g.67,488,095 C > T	−151	0%
rs2279744^e^	g.67,488,847 T > G	+601	35%

^a^From dbSNP build 128.

^b^NCBI Build 36.1.

^c^Position relative to the transcription start site (based on reference sequence mRNA).

^d^Minor allele frequencies (MAF) were calculated with 91 unrelated European individuals in this study.

^e^For comparison purposes, we included SNP309 (+601 in our nomenclature) associated with the internal promoter P2.

**Table 2:** **Most frequent *MDM2 *promoter haplotypes **

**Haplotype**	**−1494 G > A**	**40 bp deletion**	**−182 C > G**	**+309 T > G**	**Frequency**^**a**^
1A	A	No deletion	C	G	36.8%
1B	A	No deletion	C	T	17.0%
2	G	Deletion	G	T	38.5%

^a^Frequencies calculated with genotyping results of the 91 unrelated European individuals.

To assess the functional impact of the major promoter haplotypes 1 and 2, we subcloned the promoter haplotypes in the promoterless pGL3 basic Firefly luciferase reporter vector and we carried out transient transfection experiments for each haplotype-specific constructs in three cell lines (Figure 2b). Because these constructs contain only the proximal P1 promoter (rs2279744 was not included), we could not test differential promoter activities between haplotypes 1A and 1B. Significant differences were found between H1 and H2 (Figure 2a), with the promoter haplotype H1 having stronger promoter activity in all cell lines tested (Figure 2b). The relative luciferase activity driven by H1 was up to 2.3-fold higher than the luciferase levels driven by H2, indicating variable haplotype-specific expression levels of *MDM2*. The 309 G allele was only present in 1.0% of individuals carrying haplotypes other than H1A (data not shown); therefore, European individuals carrying the allele G of this SNP are more likely to have the high P1 promoter activity haplotype because of the linkage disequilibrium.

**Figure 3:**
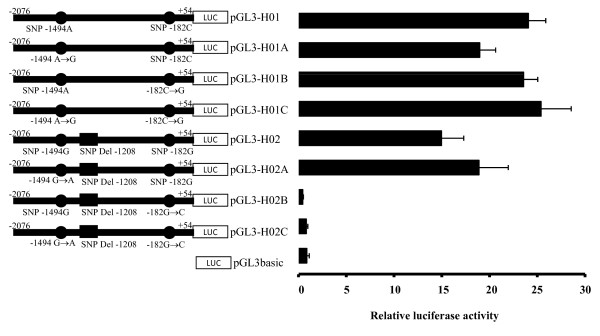
**Functional analyses of *MDM2* promoter haplotype H1- and H2-derived mutations in HeLa cells.** H1- and H2-derived constructs carrying mutations introduced by site-directed mutagenesis and tested for luciferase gene reporter assays in pGL3 basic vector (left panel). Relative luciferase activity of the H1- and H2-derived promoter haplotypes was measured following transient transfection in HeLa cells (right panel). The empty promoterless pGL3 basic vector was used as negative control. Results are expressed in a ratio of Firefly/*Renilla* activity multiplied by 100. Promoter haplotype H2 was used as reference against which relative expression was compared. The *p* values are calculated from four replicates with unpaired student’s *t* test. The *p* value between H1 and H2 is 0.0008.

Using *in silico* predictive tools, none of these SNPs seem to affect the putative binding of known transcription factors. However, the 40 bp indel contains several predicted transcription factor binding sites (data not shown). In an attempt to identify the *cis*-acting elements responsible for the observed changes in *MDM2* P1 promoter activity, we modified the allele combination in both haplotypes using site-directed mutagenesis (Figure 3). None of the allele combinations in the context of the 40 bp insertion (defining H1) significantly affected the promoter activity of the corresponding H1-derived haplotypes. In the context of the 40 bp deletion (defining H2), the −1494A > G variant (rs1144944) does not affect the H2-derived promoter activity. However, the introduction of allele −182 C (instead of allele G) completely abrogated the promoter activity when combined with the 40 bp deletion compared to the H1-derived construct. This indicates the role of the 40 bp indel variation in the observed allelic promoter activity and the presence of a putative *cis*-acting element at position −182. Taken together, these results support the functional impact of *MDM2* promoter haplotypes on the promoter activity.

## Discussion

In more than half the tumors with a fault in the p53 pathway, TP53 itself is not mutated but the p53 pathway is abrogated. Mechanisms that result in this abrogation include increased expression of MDM2 [7] and deletion or epigenetic inactivation of the p53-positive regulator and MDM2 inhibitor ADP-ribosylating factor [39,40]. MDM2 might influence cancer risk through its interaction with other key cancer genes with various functions [41-44]. The MDM2 oncogene is overexpressed in various human cancers and its expression correlates with the phenotypes of high-grade, late-stage, and resistant tumors [45,46]*.* MDM2 has an important role in cancer development, mostly through inactivation of the p53 pathway [46]*.* By contrast, the p53-independent MDM2-mediated tumorigenesis is less understood.

At the promoter level, regulation of *MDM2* expression is complex involving two promoters, P1 and P2, which govern transcripts with different translational potentials. In this report, we characterized the two major haplotypes that correspond to the upstream p53-independent constitutive P1 promoter. Unlike the p53-responsive P2 promoter, the P1 promoter lacks an identified TATA box and p53-responsive element [23,47]. We showed that the constitutive expression levels of *MDM2* might at least be partially regulated by distinct promoter SNPs, particularly the 40 bp deletion and the corresponding promoter haplotypes (see Results section). Previous work has shown a correlation of rs937282 with allelic differences in promoter activity, with the allele −182 G having high promoter activity [48]. However, in our hands, the −182 G allele was associated with the low-activity P1 promoter haplotype. This discrepancy could be explained by the fact that the extended promoter P1 haplotype was not determined in their study. The latter is particularly relevant when considering the observed impact of the SNP-182 C > G alleles in the context of the presence/absence of the 40 bp deletion.

Most previous studies have been focused on the impact of rs2279744 (SNP309 (T > G)), which is located in the p53-dependent promoter P2. *In vitro* studies have shown that the allele SNP309G increased the affinity of Sp1 transcription activator for a putative binding site and increase the steady-state levels of *MDM2*, which in turn reduced the basal p53 levels [26,49]. Although many studies have attempted to assess the association between rs2279744 and different cancer types, the data remains controversial [27,38,50]. A clear association between rs2279744 and cancer risk was reported in Asians but not in Europeans and in Africans in a meta-analysis [38]. The explanation for this observation is unclear but could be explained by genetic heterogeneity because the observed SNP309 frequencies are variable in different populations ranging from approximately 50% in Asians to 33% and 10% in Caucasians and Africans, respectively [38]. A recent meta-analysis indicates that MDM2 SNP309 serves as a tumor susceptibility marker [51]. Finally, the transcription factor influenced by rs2279744 might be cell-type specific so that this variant does not affect *MDM2* expression in certain tissues [22].

These conflicting rather than conclusive results might be explained by several reasons, including linkage disequilibrium between SNP309 and another, yet unknown, functional SNP in *MDM2*. This linkage disequilibrium could also contribute to cancer associations with SNP309 suggesting that haplotype constructions of *MDM2* pSNPs would add force to these association studies. In this report, we showed that SNP309G was associated with the high P1 promoter activity haplotype. We believe that looking at the impact of haplotypes rather than individual SNPs on promoter activity is a more suitable approach because it takes into account the putative interaction between SNPs. In conclusion, this study revealed differential constitutive P1 promoter activities, at least *in vitro*. This observation implies that individuals who carry distinct p53-independent P1 promoter haplotypes might have a modified risk for cancer development. Association studies in large patient cohorts will helps us to further determine the importance of these haplotypes in cancer.

## Competing interests

The authors declare that they have no competing interests.

## Authors’ contributions

MEL carried out most molecular genetics experiments and drafted the manuscript. MO and ML participated in some molecular studies. MEL, MO, EK, and DS contributed to the interpretation of the data. MEL, DS and EK conceived the study, and participated in its design and coordination. All authors read and approved the final manuscript.
